# Methylacetanilide

**Published:** 1894-03-03

**Authors:** 


### METHYLACETANILIDE.

This drug, which is often called exalgin, is coming rapidly into favour, and Savill[1] has published an account of his experience. Two to four grains is a usual dose. It may be given in cachets or dissolved in water, to which a little spirit has been added, or even in pure water if that is used hot, for the drug remains in solution when the water has cooled. This solution keeps well, and the author never met with any inconvenient or uncomfortable effects, nor did it cause any toxic symptoms. In all the cases in which Savill gave it, his object was to relieve pain, and on the whole he met with much success. The pain varied much in position, constancy, character, and intensity, and was usually of a neuralgic character. In some the relief was only temporary. In half the cases where the cause of the pain was persistent the dose had to be increased, and therefore this drug, like most of its class, seems liable to make the patient tolerant of it. Younger[2] has also employed methylacetanilide at Hanwell, and he also finds it very valuable for patients suffering from neuralgia; he gave doses of one to three grains. He also found six grains every four hours of great benefit in epilepsy. Marandon[3] comes to a diametrically opposite opinion upon the value of this drug, but that is probably because he gave it to patients suffering from such severe forms of mental disorder that under no circumstances could any benefit be expected. Weismayer[4] used doses probably unnecessarily large, for he sometimes gave as much as fifteen grains for a dose. When he got vomiting, giddiness, and delirium, the pulse and the temperature were not affected. He found that it is a decided analgesic for functional pains such as neuralgia, but he does not think so highly of it as he does of antipyrin. For organic pain he considers it useless.

[1] Lancet, Nov. 25, 1893. [2] Lancet, April 8, 1893. [3] Bull. de Thérap., April 30, 1893. [4] Wiener Klin. Wochenschrift, No. 9, 1893.

